# Supplementary material for: Comparative efficacy and tolerability of new-generation antidepressants for major depressive disorder in children and adolescents: protocol of an individual patient data meta-analysis
Source: BMJ Open. 2018 Jan 5;8(1):e018357. doi: 10.1136/bmjopen-2017-018357 (PMC5781225; doi:10.1136/bmjopen-2017-018357)
Supplement: Author's manuscript [file bmjopen-2017-018357.draft_revisions.pdf]

BMJ Open

BMJ Open is committed to open peer review. As part of this commitment we make the peer review history of every article we publish publicly available.

When an article is published we post the peer reviewers' comments and the authors' responses online. We also post the versions of the paper that were used during peer review. These are the versions that the peer review comments apply to.

The versions of the paper that follow are the versions that were submitted during the peer review process. They are not the versions of record or the final published versions. They should not be cited or distributed as the published version of this manuscript.

BMJ Open is an open access journal and the full, final, typeset and author-corrected version of record of the manuscript is available on our site with no access controls, subscription charges or pay-per-view fees (http://bmjopen.bmj.com).

If you have any questions on BMJ Open's open peer review process please email editorial.bmjopen@bmj.com

BMJ Open

Comparative efficacy and tolerability of new-generation antidepressants for major depressive disorder in children and adolescents: protocol of an individual patient data meta-analysis

Journal:	*BMJ Open*
Manuscript ID	bmjopen-2017-018357
Article Type:	Protocol
Date Submitted by the Author:	22-Jun-2017
Complete List of Authors:	Zhou, Xinyu; The First Affiliated Hospital of Chongqing Medical University, Department of Psychiatry<br>Cipriani, Andrea; University of Oxford, Department of Psychiatry; Warneford Hospital, Oxford Health NHS Foundation Trust<br>Furukawa, Toshi; Kyoto University, Graduate School of Medicine and School of Public Health<br>Cuijpers, Pim; VU University Amsterdam, Department of Clinical Psychology<br>Zhang, Yuqing; The First Affiliated Hospital of Chongqing Medical University, Department of Neurology<br>Hetrick, Sarah; University of Melbourne, Centre for Youth Mental Health<br>Pu, Juncai; The First Affiliated Hospital of Chongqing Medical University, Department of Neurology<br>Yuan, Shuai; The First Affiliated Hospital of Chongqing Medical University, Department of Neurology<br>Del Giovane, Cinzia; University of Modena and Reggio Emilia, Clinical and Diagnostic Medicine and Public Health<br>Xie, Peng; The First Affiliated Hospital of Chongqing Medical University, Department of Neurology; Chongqing Medical University, Institute of Neuroscience and the Collaborative Innovation Center for Brain Science
**Primary Subject Heading**:	Mental health
Secondary Subject Heading:	Mental health
Keywords:	Depression & mood disorders < PSYCHIATRY, child, adolescent, antidepressant, individual patient data meta-analysis, systematic review

SCHOLARONE™
Manuscripts

For peer review only - http://bmjopen.bmj.com/site/about/guidelines.xhtml

Comparative efficacy and tolerability of new-generation antidepressants for major depressive disorder in children and adolescents: protocol of an individual patient data meta-analysis

Xinyu Zhou,[1] Andrea Cipriani,[2,3] Toshiaki A. Furukawa,[4] Pim Cuijpers,[5] Yuqing Zhang,[6] Sarah E. Hetrick,[7] Juncai Pu,[6] Shuai Yuan,[6] Cinzia Del Giovane,[8] Peng Xie[6]

[1] Department of Psychiatry, The First Affiliated Hospital of Chongqing Medical University, Chongqing, China

[2] Department of Psychiatry, University of Oxford, Warneford Hospital, Oxford, UK

[3] Oxford Health NHS Foundation Trust, Warneford Hospital, Oxford, UK

[4] Department of Health Promotion and Human Behavior, Kyoto University Graduate School of Medicine and School of Public Health, Kyoto, Japan

[5] Department of Clinical, Neuro and Developmental Psychology, Amsterdam Public Health research institute, Vrije Universiteit Amsterdam, The Netherlands

[6] Department of Neurology, The First Affiliated Hospital of Chongqing Medical University, Chongqing, China; Institute of Neuroscience and the Collaborative Innovation Center for Brain Science, Chongqing Medical University, Chongqing, China

[7] Orygen, The National Centre of Excellence in Youth Mental Health, and the Centre of Youth Mental Health, University of Melbourne, Melbourne, Australia

[8] Department of Diagnostic, Clinical and Public Health Medicine, University of Modena and Reggio Emilia, Modena, Italy

Direct correspondence to:

For peer review only

Prof. Peng Xie, Department of Neurology, The First Affiliated Hospital of Chongqing Medical University, 1 Youyi Road, Yuzhong District, Chongqing 400016, China; E-mail: xiepeng973@126.com

Xinyu Zhou, Andrea Cipriani, and Toshiaki A. Furukawa contributed equally to this work.

Keywords: depression, child, adolescent, antidepressant, individual patient data meta-analysis, systematic review

Word count: 2598.

For peer review only

ABSTRACT

Introduction: Although previous conventional meta-analyses and network meta-analyses have provided some important findings about pharmacological treatments for children and adolescents with depressive disorders in the past decades, several questions still remain unsolved by the aggregate data from those meta-analyses. Individual participant data meta-analysis (IPD-MA) enables exploration of the impacts of individual characteristics on treatment effects, allowing matching of treatments to specific subgroups of patients. We will perform an IPD-MA to assess the efficacy and tolerability of new-generation antidepressants for major depressive disorder in children and adolescents.

Methods and analysis: We will systematically search for all double-blind randomised controlled trials (RCTs) that have compared any new-generation antidepressant with placebo for the acute treatment of major depressive disorder in children and adolescents, in the following databases: PubMed, EMBASE, the Cochrane Library, PsycINFO, Web of Science, CINAHL, LILACS, and ProQuest Dissertations. We will contact all corresponding authors of included RCTs and ask for their cooperation in this project by providing individual participant data from the original trials. The primary outcomes will include efficacy, measured as the mean change of depression symptoms by CDRS-R scale, and tolerability, measured as the proportion of patients who withdrew from the trials early due to adverse effects. The secondary outcomes will include response rates, remission rates, deterioration rate, all-cause discontinuation, suicidal-related outcomes, as well as global functioning outcome. Using the raw de-identified study data, we will use mixed-effects logistic and linear regression models to perform the IPD-MAs. The risk of bias of included studies will be assessed using the Cochrane risk of bias tool. We will also detect the publication bias and effects of non-participation of eligible studies.

Dissemination: We will publish the results in a peer-reviewed journal. This study may have considerable

For peer review only

For peer review only - http://bmjopen.bmj.com/site/about/guidelines.xhtml

implications for practice and help improve patient care.

Protocol registration: PROSPERO CRD42016051657

For peer review only

For peer review only - http://bmjopen.bmj.com/site/about/guidelines.xhtml

Strengths and limitations of this study

1. This is the first individual participant data meta-analysis (IPD-MA) comparing the efficacy and tolerability of new-generation antidepressants for major depressive disorder in children and adolescents.

2. The study will use individual patient data that can take into account within-study and between-study differences, yield more reliable estimates of treatment effects than meta-analysis of aggregate data.

3. Individual patient data meta-analysis can provide insight into the patient groups most likely to benefit from new-generation antidepressants and the most effective kinds of antidepressants.

4. The main difficulty of this study will be collecting the patient-level information from all eligible trials, for some of the original investigators may not be willing or able to share the data. Underlying publication bias and effects of non-participation of eligible studies may restrict the conclusion.

For peer review only

For peer review only - http://bmjopen.bmj.com/site/about/guidelines.xhtml

Background

Major depressive disorder (MDD) is a commonly occurring serious mental disorder, accounting for a large portion of the global burden of disease. The overall prevalence rate of depressive disorder is about 3% in children and 6% in adolescents.[1] Depressive disorder in youth is often associated with high rates of comorbid mental disorders, functional impairment, and suicide.[2-5] For young people aged 10–19 years, depressive disorders are the leading cause of health-related burden, accounting for 6–10% of the disability-adjusted life-years.[6] Early-onset depression is an important predictor of the recurrence of depressive disorders. In a naturalistic follow up study, up to 55% pediatric patients who recovered from the first episode of MDD, had a second episode within 5 years and rose to 72% within 15 years.[7]

In the past 20 years, several new-generation antidepressants have been found to be effective in the treatment of adult MDD.[8,9] However, whether to use antidepressants in children and adolescents are still matters of controversy, mainly due to concerns about efficacy and potentially increased risk of treatment-emergent suicide in those young patients.[10,11] In 2004, some worrying interpretations from a conventional meta-analysis were shown: published data suggested a favorable risk benefit profile for some selective serotonin reuptake inhibitors (SSRIs); however, addition of unpublished data indicated that risks could outweigh the benefits of these drugs (except fluoxetine) for the treatment of depression in children and young people.[12] Recently, our published network meta-analysis showed that most currently available antidepressants do not seem to offer a clear advantage over placebo for depression in children and adolescents, and fluoxetine is probably the best option to consider when a pharmacological treatment is indicated.[13] Nevertheless, several questions still remain unsolved by the aggregate data from conventional and network meta-analyses. First, the effect sizes of some antidepressants in previous meta-analyses had large confidence/credible interval with its upper limit close to the point of no difference, which raises the question of whether this estimate is robust enough to inform clinical practice.[14] Second, most studies included both children and adolescents, but they did not separately report the data of different age groups. Thus, it remains unclear whether the antidepressants are efficacious across the diverse populations included. Third, there was strict range of baseline severity scores included in these previous meta-analyses. For example, in our previous NMA analysis, most studies focused on samples with moderate to severe depressive severity, with few trials of those with mild to moderate, or very severe range. Therefore, whether the antidepressants have similar efficacy for

For peer review only - http://bmjopen.bmj.com/site/about/guidelines.xhtml

mildly or severely depressed patients is another important question that remains. Fourth, RCTs evaluating antidepressant treatments in children and adolescents seldom report the number of patients who deteriorated during treatment, thus it is not possible to investigate mean deterioration effects found in randomized trials and its moderators using conventional and network meta-analytical approaches.

Individual participant data meta-analysis (IPD-MA) is an increasingly popular approach for synthesizing and investigating treatment effect estimates. IPD-MA has many statistical and clinical advantages over meta-analyses of aggregate data. For example, clinical heterogeneity can be reduced by controlling for patient-level covariates in IPD-MA,[15,16] which offers the potential to explore additional, more thorough, and potentially more appropriate analyses compared to those possible with aggregate data.[17] IPD-MA also provides unique opportunities to identify underlying individual characteristics as prognostic factors or negative effects across several studies.[18] Therefore, we will perform an IPD-MA to assess the efficacy and tolerability of new-generation antidepressants for major depressive disorder in children and adolescents.

Methods

Criteria for included studies

Types of studies

Studies included in this IPD-MA will be double-blind randomised controlled trials (RCTs), including studies with cluster or cross-over designs. Given possible carry-over effects, we will only consider data from the first study period in cross-over trials. We will exclude trials employing inappropriate randomisation strategies, such as quasi-randomised designs.

Types of participants

Studies will be included in the IPD-MA if they aim at (1) children and adolescents aged between 6–18 years when initially enrolled in the studies, (2) with primary diagnosis of major depressive disorder according to standard diagnostic criterion, such as the Diagnostic and Statistical Manual of Mental Disorders (DSM),[19-23] or the International Classification of Diseases (ICD).[24,25] Studies will be excluded if they included patients with bipolar depression, or treatment-resistant depression, while patients with comorbid general psychiatric disorders, such as anxiety disorder, will not be excluded.

For peer review only - http://bmjopen.bmj.com/site/about/guidelines.xhtml

Types of interventions

We will include all RCTs comparing any new-generation antidepressant with placebo during the acute treatment phase of depression in children and adolescents. The following new-generation antidepressants using prescribed oral and therapeutic dose range will be included.[8,13,26]

1. Selective serotonin reuptake inhibitors (SSRIs), e.g., fluoxetine, fluvoxamine, paroxetine, sertraline, citalopram, and escitalopram.

2. Serotonin and norepinephrine reuptake inhibitors (SNRIs), e.g., duloxetine, venlafaxine, desvenlafaxine, milnacipran, levomilnacipran.

3. Other antidepressants, e.g., mirtazapine, mianserin, nefazodone, trazodone, vortioxetine, vilazodone, bupropion, reboxetine, and agomelatine.

We will only include RCTs with a minimum of 4-week treatment duration, because the onset of benefit for most antidepressants often takes at least 4 weeks.[27] We will exclude trials designed as maintenance treatment or relapse prevention, unless outcome data from the acute phase can be accessed separately. Combination studies and augmentation studies (e.g. combined with different antidepressant or psychotherapy) will also be excluded.

Types of outcome measures

Primary outcomes

(1) Overall efficacy

The primary outcome of efficacy will be the overall change in depressive symptoms, as measured using Children's Depression Rating Scale Revised (CDRS-R[28]) from baseline to endpoint. For RCTs which didn't measure CDRS-R, we will try to convert other depression scales (such as HAMD[29] or MADRS[30]) scores to CDRS-R scores, by using a factor derived from the RCTs that used both scales.

As shown in our previous network meta-analysis,[13] trial duration varied from 6 weeks to 36 weeks, and the majority of trials employed a treatment duration of 8 weeks. We will try to obtain repeated measures from individual trials if possible. To improve comparability between the included trials, we will prefer the data from 8-week (or the closest to 8-week) time point for efficacy outcomes.

(2) Overall tolerability

The tolerability of treatment will be the proportion of patients who drop out of the trials early due to side

For peer review only - http://bmjopen.bmj.com/site/about/guidelines.xhtml

effects at the end of the blinded treatment.

Secondary outcomes

(1) Response rate

Response rate will be defined as 50% reduction from baseline to endpoint on CDRS-R (or another standardised rating scale such as HAMD or MADRS).

(2) Remission rate

Remission rate will be defined as the CDRS scores of less than 28.[31]

(3) Deterioration rate

Deterioration represents the depression symptom severity increases after treatment. Deterioration rate will be defined as the proportion of patients whose CDRS-R scores from baseline to endpoint had reliable change index below the cut-off of −1.96.[32]

(4) Overall acceptability

The acceptability of treatment will be the proportion of patients who drop out of the trials early for any cause at the end of the blinded treatment.

(5) Suicide-related outcomes

Suicide-related dichotomous and continuous outcomes will be measured. We will extract the number of participants with suicide-related events (combined suicidal ideation and suicidal behavior) during the acute treatment, as measured on a standardised, validated and reliable rating scale, or reported cases of suicidal ideation and behavior.[33] In addition, if data are available, we will also collect data on suicidal ideation as a continuous outcome where a standardised, validated, and reliable rating scale, such as the Suicidal Ideation Questionnaire-Junior High School version (SIQ-JR),[34] has been used.

(6) Global functioning

The outcome of global functioning will be the overall change in validated scales from baseline to endpoint. The commonly used tools of functioning scales included the Children's Global Assessment Scale (CGAS),[35] Global Assessment of Functioning (GAF),[36] etc.

Data Sources and Search strategy

We will first include the RCTs identified by the criteria used in our previous work.[13,37] Then we will update the extensive searching to bring it up to date. Briefly, we will identify any published and unpublished

For peer review only - http://bmjopen.bmj.com/site/about/guidelines.xhtml

For peer review only

RCTs, in any language, from electronic systematic searches of PubMed, EMBASE, the Cochrane Library, PsycINFO, Web of Science, LILACS, CINAHL, and ProQuest Dissertations. Electronic databases will be searched with free words and Medical Subject Headings (MeSH) terms using the following strategy: [depress* or dysthymi* or mood disorder* or affective disorder*], combined with [adolesc* or child* or boy* or girl* or juvenil* or minors or paediatri* or pediatri* or pubescen* or school* or student* or teen* or young or youth*], and combined with a list of antidepressants, including [selective serotonin reuptake inhibitors or SSRI or fluoxetine or fluvoxamine or paroxetine or sertraline or citalopram or escitalopram or serotonin and norepinephrine reuptake inhibitors or SNRI or duloxetine or venlafaxine or desvenlafaxine or milnacipran or levomilnacipran or mirtazapine or mianserin or nefazodone or trazodone or vortioxetine or vilazodone or bupropion or reboxetine or agomelatine]. In addition, we will also identify additional trials and unpublished data by searching: (1) international trials registries, mainly including of ClinicalTrials.gov, and World Health Organization International Clinical Trials Registry Platform (ICTRP); (2) USA Food and Drug Administration (FDA) reports; (3) websites of main manufactures, e.g., GlaxoSmithKline, Lilly, Organon, Forest Pharmaceuticals, Bristol-Myers Squibb; (3) manual hand search of key journals and conference proceedings, e.g., *J Child Adolesc Psychopharmacol, J Am Acad Child Adolesc Psychiatry, Child Adolesc Psychiatry Ment Health, Psychopharmacol Bull, Arch Gen Psychiatry, Am J Psychiatry, Eur Psychiatry, Depress Anxiety*. Additional relevant RCTs will be obtained by hand-searching reference lists of included studies and relevant reviews. We will also contact corresponding authors of included RCTs, manufactures, FDA, and other possible institutions for unpublished trials.

Study selection and data extraction

Selection of trials

We will first manually remove duplicates of initial search results. Then two experienced reviewers will independently screen titles and abstracts from the retrieved results for possible candidates. We will exclude the trials in which both reviewers judge they do not meet eligibility criteria. Full texts of all remaining papers will be retrieved, and two reviewers will independently examine whether to include them by the same eligibility criteria. Any difference of opinion, for each step, between the reviewers will be resolved through discussion with another member of the reviewing team, or by contacting the authors of the trials for clarification. The selection process of retrieved studies and the reasons for exclusion of trials (e.g. ineligible populations, not randomized trials) will be shown in a flow chart.

For peer review only - http://bmjopen.bmj.com/site/about/guidelines.xhtml

For peer review only

Data collection

From the included RCTs, two reviewers will independently extract the trial level information using standardized data collection forms, including trial characteristics, patient characteristics, intervention details, and any other information relevant to this review.

We will contact all corresponding authors of included RCTs and ask for their cooperation in this project. The corresponding authors' contact information will be abstracted from the papers, online research profiles (e.g., Google Scholar), or other available ways. Specifically, we will (1) send emails to the authors explaining the study purpose, and invite them to cooperate in this project; (2) send reminder emails 4 and 6 weeks later if no response; (3) contact the corresponding authors by phone or possible personal contacts.

Trial level information and individual participant data to be obtained from the original authors are shown in Table 1, respectively. The raw data can be provided in any convenient manner (such as by email) in common types of electronic format, such as Excel, SPSS, Stata, etc. All obtained data will be converted to a uniform format, and saved on a secure server at the Chongqing Medical University. The data set will not contain any personal identifier of patients, such as names or phone numbers. Only authorised members of the research team will be allowed to access the data set.

Data checking

We will check for data-entry mistakes and consistency, and reanalyse the data within each study according to the original statistical methodology, the results will be compared with the published summary results. Any error will be resolved by discussion with the original investigators, and data corrections will be made if necessary.

Missing data

Handling of missing data will depend on the proportion of missing data in the full dataset. In general, we will prefer to manage missing data for both patient characteristics and outcomes through multiple imputation methods, such as multiple imputation (MI) and mixed-effects model repeated measures (MMRM), because multiple imputation techniques with a missing at random assumption tends to yield more unbiased results than single imputation methods.[38] Missing data will be imputed using the command *mi impute mvn* in Stata version 14.0. However, if we obtain repeated measures from individual trials, we will use MMRM approach.

For peer review only - http://bmjopen.bmj.com/site/about/guidelines.xhtml

Risk of bias assessment and quality of study

Two independent review authors will use the Cochrane Collaboration's Risk of bias' tool[39] to evaluate the methodological and hence bias risk of eligible studies, and quality assessment will be reported on a study level. The risk of bias will be assessed across seven items, including random sequence generation, allocation concealment, blinding of intervention, blinding of outcome assessment, incomplete outcome data, selective outcome reporting, and other bias, with three level of risk (high, unclear, low). We will rate the quality of study as follows: high risk study (2 or more items rated as high risk of bias); low risk study (5 or more items rated as low risk and no more than one as high risk); unclear risk study (all remaining situations). Any disagreements will be resolved by consensus or consulting the original authors.

Publication bias and effects of non-participation of eligible studies

We will use contour enhanced funnel plot to detect publication bias for study level data (full set of studies meeting inclusion criteria) and patient level data (the set of studies that were included in the IPD-MA), if at least ten studies are available.[40] We will also use Egger's test to quantify the bias, with a P value < 0.10 taken to indicate statistical evidence of asymmetry.[41] In order to examine the effects of non-participation of eligible studies, we will conduct a meta-regression analysis with the effect size of primary outcomes (based on study level data) as the dependent variables, and whether or not the patient level data are included as the predictor indicating. The analyses will be conducted in Stata version 14.0.

Statistical analysis

All analyses will be performed by intention-to-treat (ITT) analysis. Descriptive statistics will be presented as mean (SD) or median (IQR) for continuous variables and number (percent) for categorical variables.

Individual patient data meta-analyses

We will first use the one-stage approach to perform the IPD-MAs, as it offers the highest degree of flexibility for making necessary assumptions,[42] and uses a more exact statistical approach than two-stage approach.[43] We will perform analyses in Stata with the commands *mixed* (for linear random-effects models), *meqrlogit* (for logistic models) and *ipdforest* (for forest plot).[44] To account for between study differences, we will use mixed-effects logistic models for categorical outcomes, and mixed-effects linear regression models for

For peer review only - http://bmjopen.bmj.com/site/about/guidelines.xhtml

continuous outcomes. Treatment assignment will be introduced as a fixed-effects variable "treatment". As outcomes might vary across studies, we will force the "study" and the interaction term "study*treatment" as random-effects variables into all models. The important clinical and demographic predictors variables (e.g., sex[45], age[46], baseline severity score[47], and treatment duration) will be used as regressors in the models. The heterogeneity of treatment effects across studies will be assessed using the I^2 statistic.[48]

Ethics and dissemination

This protocol is registered in the International Prospective Register of Systematic Reviews (PROSPERO) at the National Health Service Centre for Reviews and Dissemination at the University of York (Registration number: CRD42016051657). No ethics review is required for this IPD meta-analysis, since informed consent has already been obtained from the patients by the trial investigators before the trial was conducted. We will publish the results in a peer-reviewed journal.

For peer review only

For peer review only - http://bmjopen.bmj.com/site/about/guidelines.xhtml

Contributors PX and XZ conceived the study and drafted the protocol. PX and XZ wrote the first draft of the manuscript. AC, TAF, PC, SEH, CDG assisted in protocol design and revision. XZ, YZ, JP and SY participated in the search strategy development. CDG, AC and TAF participated in the design of data synthesis and analysis. All the authors have approved the publication of the protocol.

Funding This work is supported by the National Basic Research Program of China (973 Program) (Grant No. 2009CB918300). The funders had no role in the protocol design; the writing of the protocol; or the decision to submit the protocol for publication.

Competing interests AC reports personal fees from Accord Healthcare as an expert witness for a patent issue about quetiapine extended release. TAF has received lecture fees from Eli Lilly, Janssen, Meiji, MSD, Otsuka, Pfizer and Tanabe-Mitsubishi, and consultancy fees from Sekisui Chemicals and Takeda Science Foundation. He has received royalties from Igaku-Shoin and Nihon Bunka Kagaku-sha publishers. He has received research support from Mochida and Tanabe-Mitsubishi. SEH is an Editor of the Cochrane Common Mental Disorders Group, an author of the Cochrane systematic review of newer generation antidepressants for depression in children and adolescents, and an author (senior) on the Cochrane review of psychological, pharmacological and their combination for child/adoles depression. XZ, PC, YZ, JP, SY, CDG, and PX declare no competing interests.

Acknowledgments Andrea Cipriani is supported by the National Institute for Health Research Oxford Cognitive Health Clinical Research Facility.

Provenance and peer review Not commissioned; externally peer-reviewed.

Open Access This is an Open Access article distributed in accordance with the Creative Commons Attribution- Non Commercial (CC BY-NC 4.0) license, which permits others to distribute, remix, adapt, build upon this work non-commercially, and license their derivative works on different terms, provided the

For peer review only - http://bmjopen.bmj.com/site/about/guidelines.xhtml

original work is properly cited and the use is non-commercial. See:

http://creativecommons.org/licenses/by-nc/4.0/

For peer review only

For peer review only - http://bmjopen.bmj.com/site/about/guidelines.xhtml

References

1. Jane Costello E, Erkanli A, Angold A. Is there an epidemic of child or adolescent depression? *J Child Psychol Psychiatry* 2006;47(12):1263-71.
2. Hawton K, Saunders KE, O'Connor RC. Self-harm and suicide in adolescents. *Lancet* 2012;379(9834):2373-82.
3. Rohde P, Lewinsohn PM, Klein DN, *et al*. Key characteristics of major depressive disorder occurring in childhood, adolescence, emerging adulthood, adulthood. *Clin Psychol Sci* 2013;1(1):41-53.
4. Jaffee SR, Moffitt TE, Caspi A, *et al*. Differences in early childhood risk factors for juvenile-onset and adult-onset depression. *Arch Gen Psychiatry* 2002;59(3):215-22.
5. Siu AL, U.S. Preventive Services Task Force. Screening for depression in children and adolescents: U.S. Preventive Services Task Force recommendation statement. *Ann Intern Med* 2016; 164(5):360-6.
6. Gore FM, Bloem PJ, Patton GC, *et al*. Global burden of disease in young people aged 10-24 years: a systematic analysis. *Lancet* 2011;377(9783):2093-102.
7. Kovacs M, Obrosky S, George C. The course of major depressive disorder from childhood to young adulthood: recovery and recurrence in a longitudinal observational study. *J Affect Disord* 2016;203:374-81.
8. Cipriani A, Furukawa TA, Salanti G, *et al*. Comparative efficacy and acceptability of 12 new-generation antidepressants: a multiple-treatments meta-analysis. *Lancet* 2009;373(9665):746-58.
9. Kennedy SH, Lam RW, Mcintyre RS, *et al*. Canadian Network for Mood and Anxiety Treatments (CANMAT) 2016 clinical guidelines for the management of adults with major depressive disorder: section 3. Pharmacological treatments. *Can J Psychiatry* 2016;61(9):540-60.
10. Bridge JA, Iyengar S, Salary CB, *et al*. Clinical response and risk for reported suicidal ideation and suicide attempts in pediatric antidepressant treatment: a meta-analysis of randomized controlled trials. *JAMA* 2007;297(15):1683-96.
11. U.S. Food and Drug Administration (FDA). Suicidality in children and adolescents being treated with antidepressant medications. www.fda.gov/drugs/drugsafety/postmarketdrugsafetyinformationforpatientsandproviders/ucm161679.htm (accessed 21 Jun 2017).
12. Whittington CJ, Kendall T, Fonagy P, *et al*. Selective serotonin reuptake inhibitors in childhood depression: systematic review of published versus unpublished data. *Lancet* 2004;363(9418):1341-5.
13. Cipriani A, Zhou X, Del Giovane C, *et al*. Comparative efficacy and tolerability of antidepressants for

For peer review only - http://bmjopen.bmj.com/site/about/guidelines.xhtml

major depressive disorder in children and adolescents: a network meta-analysis. *Lancet* 2016;388(10047):881-90.

14. Salanti G, Del Giovane C, Chaimani A, *et al*. Evaluating the quality of evidence from a network meta-analysis. *PLoS One* 2014;9(7):e99682.
15. Riley RD, Lambert PC, Abo-Zaid G. Meta-analysis of individual participant data: rationale, conduct, and reporting. *BMJ* 2010;340(7745):521-25.
16. Debray TP, Moons KG, Ahmed I, *et al*. A framework for developing, implementing, and evaluating clinical prediction models in an individual participant data meta-analysis. *Stat Med* 2013;32(18):3158-80.
17. Tudur Smith C, Marcucci M, Nolan SJ, *et al*. Individual participant data meta-analyses compared with meta-analyses based on aggregate data. *Cochrane Database Syst Rev* 2016, 9:MR000007.
18. Debray TP, Riley RD, Rovers MM, *et al*. Individual participant data (IPD) meta-analyses of diagnostic and prognostic modeling studies: guidance on their use. *PLoS Med* 2015;12(10):e1001886.
19. American Psychiatric Association. *Diagnostic and statistical manual of mental disorders (DSM-III)*. 3rd Edition. Washington DC: American Psychiatric Association, 1980.
20. American Psychiatric Association. *Diagnostic and statistical manual of mental disorders (DSM-III-R)*. 3rd Edition. Washington DC: American Psychiatric Association, 1987.
21. American Psychiatric Association. *Diagnostic and statistical manual of mental disorders (DSM-IV)*. 4th Edition. Washington DC: American Psychiatric Association, 1994.
22. American Psychiatric Association. *Diagnostic and statistical manual of mental disorders (DSM-IV-TR)*. 4th Edition. Washington DC: American Psychiatric Association, 2000.
23. American Psychiatric Association. *Diagnostic and statistical manual of mental disorders (DSM-5)*. 5th Edition. Arlington, VA: American Psychiatric Publishing, 2013.
24. World Health Organization (WHO). *The Ninth Revision of the International Classification of Diseases and Related Health Problems (ICD-9)*. Geneva: World Health Organization, 1978.
25. World Health Organization. *The ICD-10 classification of mental and behavioural disorders: clinical description and diagnostic guidelines*. Geneva: World Health Organization, 1992.
26. Hetrick SE, McKenzie JE, Cox GR, *et al*. Newer generation antidepressants for depressive disorders in children and adolescents. *Cochrane Database Syst Rev* 2012;11(11):CD004851.
27. American Psychiatric Association. *Guideline watch: practice guideline for the treatment of patients with major depressive disorder*. 3nd edn.

For peer review only - http://bmjopen.bmj.com/site/about/guidelines.xhtml

http://psychiatryonline.org/pb/assets/raw/sitewide/practice_guidelines/guidelines/mdd.pdf (accessed 21 Jun 2017).

28. Poznanski EO, Mokros HB. *Children's depression rating scale, revised (CDRS-R): manual.* Los Angeles, CA: Western Psychological Services, 1996.

29. Hamilton M. A rating scale for depression. *J Neurol Neurosurg Psychiatry* 1960;23:56-62.

30. Montgomery SA, Asberg M. A new depression scale designed to be sensitive to change. *Br J Psychiatry* 1979;134:382-9.

31. Emslie GJ, Rush AJ, Weinberg WA, *et al*. A double-blind, randomized, placebo-controlled trial of fluoxetine in children and adolescents with depression. *Arch Gen Psychiatry* 1997; 54(11):1031-7.

32. Jacobson N, Truax P. Clinical significance: a statistical approach to defining meaningful change in psychotherapy research. *J Consult Clin Psychol* 1991;59(1):12-9.

33. U.S. Food and Drug Administration (FDA). *Guidance for industry: suicidal ideation and behavior: prospective assessment of occurrence in clinical trials. Draft guidance.* 1st revision. http://www.fda.gov/drugs/guidancecomplianceregulatoryinformation/guidances/ucm315156.htm (accessed 21 Jun 2017).

34. Reynolds WM. *Professional Manual for the Suicidal Ideation Questionnaire*. Odessa, FL: Psychological Assessment Resources, 1988.

35. Shaffer D, Gould MS, Brasic J, *et al*. A children's global assessment scale (CGAS). *Arch Gen Psychiatry* 1983;40(11):1228-31.

36. Endicott J, Spitzer RL, Fleiss JL, *et al*. The global assessment scale: a procedure for measuring overall severity of psychiatric disturbance. *Arch Gen Psychiatry* 1976;33(6):766-71.

37. Zhou X, Qin B, Whittington C, *et al*. Comparative efficacy and tolerability of first-generation and newer-generation antidepressant medications for depressive disorders in children and adolescents: study protocol for a systematic review and network meta-analysis. *BMJ Open* 2015;5(9):e007768.

38. DondersAR, van der Heijden GJ, Stijnen T, *et al*. Review: a gentle introduction to imputation of missing values. *J Clin Epidemiol* 2006;59(10):1087-91.

39. Higgins JPT, Green S. *Cochrane handbook for systematic reviews of interventions version 5.1. 0* [updated March 2011]. The Cochrane Collaboration, 2011. http://handbook.cochrane.org/ (accessed 21 Jun 2017).

40. Sterne JA, Sutton AJ, Ioannidis JP, *et al*. Recommendations for examining and interpreting funnel plot asymmetry in meta-analyses of randomised controlled trials. *BMJ* 2011;343:d4002.

For peer review only - http://bmjopen.bmj.com/site/about/guidelines.xhtml

41. Egger M, Davey Smith G, Schneider M, *et al*. Bias in meta-analysis detected by a simple, graphical test. *BMJ* 1997;315(7129):629-34.

42. Debray TP, Moons KG, van Valkenhoef G, *et al*. Get real in individual participant data (IPD) meta-analysis: a review of the methodology. *Res Synth Methods* 2015;6(4):293-309.

43. Debray TP, Moons KG, Abo-Zaid GM, *et al*. Individual participant data meta-analysis for a binary outcome: one-stage or two-stage? *PLoS One* 2013;8(4):e60650.

44. Kontopantelis E, Reeves D. A short guide and a forest plot command (ipdforest) for one-stage meta-analysis. *Stata J* 2013;13(3):574-87.

45. Sotsky SM, Glass DR, Shea MT, *et al*. Patient predictors of response to psychotherapy and pharmacotherapy: findings in the NIMH Treatment of Depression Collaborative Research Program. *Am J Psychiatry* 1991;148(8):997-1008.

46. Kowatch RA, Carmody TJ, Emslie GJ, *et al*. Prediction of response to fluoxetine and placebo in children and adolescents with major depression: a hypothesis generating study. *J Affect Disord* 1999; 54(3):269-76.

47. Curry J, Rohde P, Simons A, *et al*. Predictors and moderators of acute outcome in the Treatment for Adolescents With Depression Study (TADS). *J Am Acad Child Adolesc Psychiatry 2006*, 45(12):1427-39.

48. Higgins JP, Thompson SG, Deeks JJ, *et al*. Measuring inconsistency in meta-analyses. *BMJ* 2003;327(7414):557-60.

For peer review only

For peer review only - http://bmjopen.bmj.com/site/about/guidelines.xhtml

TABLES

Table 1. Data items to be requested for individual participant data meta-analysis

Trial level information	Demographic and baseline characteristics	Therapeutic process	Outcomes
1. Study protocol<br>2. Clinical study report (if available);<br>3. List of publications<br>4. Setting (such as primary care, hospitals, clinics)<br>5. Information about the risk of bias (sequence generation, allocation of concealment, masking, and ITT analysis)<br>6. Any other information relevant to this review	1. Unique identification number for anonymity<br>2. Date of randomization<br>3. Sex (Male, female)<br>4. Race (White/Caucasian, African/African-American, Asian, Multiracial, Other)<br>5. Body mass index (BMI), kg/m^2<br>6. Height, cm<br>7. Weight, kg<br>8. Age, year<br>9. Age at onset, year<br>10. Length of illness, month<br>11. Number of MDD episodes<br>12. Duration of current episode, month<br>13. Baseline depression symptom score<br>14. Baseline quality of life and functioning score<br>15. Previous and/or ongoing secondary psychiatric disorder (anxiety disorder, externalizing disorder (ADHD, conduct disorder, oppositional defiant disorder), psychotic disorder, substance-related disorder)<br>16. Family history of MDD<br>17. Household (Two parents, other)<br>18. Number of siblings<br>19. Life and social history<br>20. Previous suicide attempt	1. Treatment (antidepressant, placebo)<br>2. Dose range<br>3. Total actual drug exposure<br>4. Treatment duration<br>5. Co-prescriptions other than antidepressant<br>6. Prior treatments (no therapy, psychotherapy, pharmacotherapy, both psychotherapy and pharmacotherapy)	1. Depression symptom scores at each evaluation (scale, time point)<br>2. Quality of life and functioning scores at each evaluation (scale, time point)<br>3. Study discontinuation and reason (dropout before starting the treatment, lack of efficacy, adverse events, others)<br>4. Adverse events<br>5. Serious adverse events (SAEs)<br>6. Suicide-related event

For peer review only

For peer review only - http://bmjopen.bmj.com/site/about/guidelines.xhtml

PRISMA-P (Preferred Reporting Items for Systematic review and Meta-Analysis Protocols) 2015 checklist: recommended items to address in a systematic review protocol*

Section and topic	Item No	Checklist item	Reported on Page #
**ADMINISTRATIVE INFORMATION**			
Title:			
Identification	1a	Comparative efficacy and tolerability of new-generation antidepressants for major depressive disorder in children and adolescents: protocol of an individual patient data meta-analysis	1
Update	1b	None	
Registration	2	PROSPERO CRD42016051657	4
Authors:			
Contact	3a	Xinyu Zhou (Department of Psychiatry, The First Affiliated Hospital of Chongqing Medical University, Chongqing, China)<br>Yuqing Zhang, Juncai Pu, Shuai Yuan, Peng Xie (Department of Neurology, The First Affiliated Hospital of Chongqing Medical University, Chongqing, China; Institute of Neuroscience and the Collaborative Innovation Center for Brain Science, Chongqing Medical University, Chongqing, China)<br>Andrea Cipriani (Department of Psychiatry, University of Oxford, Warneford Hospital, Oxford, UK)<br>Toshiaki A. Furukawa (Department of Health Promotion and Human Behavior, Kyoto University Graduate School of Medicine and School of Public Health, Kyoto, Japan)<br>Pim Cuijpers (Department of Clinical, Neuro and Developmental Psychology, Amsterdam Public Health research institute, Vrije Universiteit Amsterdam, The Netherlands)<br>Sarah E. Hetrick (Orygen, The National Centre of Excellence in Youth Mental Health, and the Centre of Youth Mental Health, University of Melbourne, Melbourne, Australia)<br>Cinzia Del Giovane (Department of Diagnostic, Clinical and Public Health Medicine, University of Modena and Reggio Emilia, Modena, Italy)<br><br>Corresponding author :Peng Xie, Department of Neurology, The First Affiliated Hospital of Chongqing Medical University, 1 Youyi Road, Yuzhong District, Chongqing 400016, China; E-mail: xiepeng973@126.com	1-2
Contributions	3b	PX and XZ conceived the study and drafted the protocol. PX and XZ wrote the first draft of the manuscript. AC, TAF, PC, SEH, CDG assisted in protocol design and revision. XZ, YZ, JP and SY participated in the search strategy development. CDG, AC and TAF participated in the design of data synthesis and analysis. All the authors have approved the publication of the protocol.	14
Amendments	4	None	
Support:			
Sources	5a	National Basic Research Program of China (973 Program) (Grant No. 2009CB918300)	14

For peer review only

Sponsor	5b	This work is supported by the National Basic Research Program of China (973 Program) (Grant No. 2009CB918300).	
Role of sponsor or funder	5c	The funders had no role in the protocol design; the writing of the protocol; or the decision to submit the protocol for publication.	
**INTRODUCTION**			
Rationale	6	Depressive disorder in children and adolescents is a major public health problem. The course of depressive disorder in young people is often characterised by protracted episodes, frequent recurrence, and comorbid psychiatric disorders. In the past 20 years, several new-generation antidepressants have been found to be effective in the treatment of adult MDD. However, whether to use antidepressants in children and adolescents are still matters of controversy, mainly due to concerns about efficacy and potentially increased risk of treatment-emergent suicide in those young patients. Recently, our published network meta-analysis showed that most currently available antidepressants do not seem to offer a clear advantage over placebo for depression in children and adolescents, and fluoxetine is probably the best option to consider when a pharmacological treatment is indicated.13 Nevertheless, several questions still remain unsolved by the aggregate data from conventional and network meta-analyses. Individual participant data meta-analysis (IPD-MA) is an increasingly popular approach for synthesizing and investigating treatment effect estimates. IPD-MA has many statistical and clinical advantages over meta-analyses of aggregate data.	6-7
Objectives	7	Therefore, we will perform an IPD-MA to assess the efficacy and tolerability of new-generation antidepressants for major depressive disorder in children and adolescents.	7
**METHODS**			
Eligibility criteria	8	**Types of participants**<br>Studies will be included in the IPD-MA if they aim at (1) children and adolescents aged between 6–18 years when initially enrolled in the studies, (2) with primary diagnosis of major depressive disorder according to standard diagnostic criterion, such as the Diagnostic and Statistical Manual of Mental Disorders (DSM), or the International Classification of Diseases (ICD). Studies will be excluded if they included patients with bipolar depression, or treatment-resistant depression, while patients with comorbid general psychiatric disorders, such as anxiety disorder, will not be excluded.<br><br>**Types of studies**<br>Studies included in this IPD-MA will be double-blind randomised controlled trials (RCTs), including studies with cluster or cross-over designs. Given possible carry-over effects, we will only consider data from the first study period in cross-over trials. We will exclude trials employing inappropriate randomisation strategies, such as quasi-randomised designs.<br><br>**Types of interventions**<br>We will include all RCTs comparing any new-generation antidepressant with placebo during the acute treatment phase of depression in children and adolescents. The following new-generation antidepressants using prescribed oral and therapeutic dose range will be included.<br>1. Selective serotonin reuptake inhibitors (SSRIs), e.g., fluoxetine, fluvoxamine, paroxetine, sertraline, citalopram, and escitalopram.<br>2. Serotonin and norepinephrine reuptake inhibitors (SNRIs), e.g., duloxetine, venlafaxine, desvenlafaxine, milnacipran,	7-8

For peer review only

		levomilnacipran.<br>3. Other antidepressants, e.g., mirtazapine, mianserin, nefazodone, trazodone, vortioxetine, vilazodone, bupropion, reboxetine, and agomelatine.<br>We will only include RCTs with a minimum of 4-week treatment duration, because the onset of benefit for most antidepressants often takes at least 4 weeks. We will exclude trials designed as maintenance treatment or relapse prevention, unless outcome data from the acute phase can be accessed separately. Combination studies and augmentation studies (e.g. combined with different antidepressant or psychotherapy) will also be excluded.	
Information sources	9	We will first include the RCTs identified by the criteria used in our previous work. Then we will update the extensive searching to bring it up to date. Briefly, we will identify any published and unpublished RCTs, in any language, from electronic systematic searches of PubMed, EMBASE, the Cochrane Library, PsycINFO, Web of Science, LILACS, CINAHL, and ProQuest Dissertations. In addition, we will also identify additional trials and unpublished data by searching: (1) international trials registries, mainly including of ClinicalTrials.gov, and World Health Organization International Clinical Trials Registry Platform (ICTRP); (2) USA Food and Drug Administration (FDA) reports; (3) websites of main manufactures, e.g., GlaxoSmithKline, Lilly, Organon, Forest Pharmaceuticals, Bristol-Myers Squibb; (3) manual hand search of key journals and conference proceedings, e.g., J Child Adolesc Psychopharmacol, J Am Acad Child Adolesc Psychiatry, Child Adolesc Psychiatry Ment Health, Psychopharmacol Bull, Arch Gen Psychiatry, Am J Psychiatry, Eur Psychiatry, Depress Anxiety. Additional relevant RCTs will be obtained by hand-searching reference lists of included studies and relevant reviews. We will also contact corresponding authors of included RCTs, manufactures, FDA, and other possible institutions for unpublished trials.	9-10
Search strategy	10	[depress* or dysthymi* or mood disorder* or affective disorder*], combined with [adolesc* or child* or boy* or girl* or juvenil* or minors or paediatri* or pediatri* or pubescen* or school* or student* or teen* or young or youth*], and combined with a list of antidepressants, including [selective serotonin reuptake inhibitors or SSRI or fluoxetine or fluvoxamine or paroxetine or sertraline or citalopram or escitalopram or serotonin and norepinephrine reuptake inhibitors or SNRI or duloxetine or venlafaxine or desvenlafaxine or milnacipran or levomilnacipran or mirtazapine or mianserin or nefazodone or trazodone or vortioxetine or vilazodone or bupropion or reboxetine or agomelatine].	10
Study records:			
Data management	11a	We will first manually remove duplicates of initial search results. We will screening citations based on the inclusion and exclusion criteria.	10
Selection process	11b	Then two experienced reviewers will independently screen titles and abstracts from the retrieved results for possible candidates. We will exclude the trials in which both reviewers judge they do not meet eligibility criteria. Full texts of all remaining papers will be retrieved, and two reviewers will independently examine whether to include them by the same eligibility criteria. Any difference of opinion, for each step, between the reviewers will be resolved through discussion with another member of the reviewing team, or by contacting the authors of the trials for clarification. The selection process of retrieved studies and the reasons for exclusion of trials (e.g. ineligible populations, not randomized trials) will be shown in a flow chart.	10
Data collection process	11c	From the included RCTs, two reviewers will independently extract the trial level information using standardized data collection forms, including trial characteristics, patient characteristics, intervention details, and any other information relevant to this review. We will contact all corresponding authors of included RCTs and ask for their cooperation in this project. The corresponding authors' contact information will be abstracted from the papers, online research profiles (e.g., Google Scholar), or other available	11

		ways. Specifically, we will (1) send emails to the authors explaining the study purpose, and invite them to cooperate in this project; (2) send reminder emails 4 and 6 weeks later if no response; (3) contact the corresponding authors by phone or possible personal contacts.	
Data items	12	Trial level information and individual participant data to be obtained from the original authors are shown in Table 1, respectively.	11, 20
Outcomes and prioritization	13	Types of outcome measures<br>Primary outcomes<br>(1) Overall efficacy<br>The primary outcome of efficacy will be the overall change in depressive symptoms, as measured using Children's Depression Rating Scale Revised (CDRS-R) from baseline to endpoint. For RCTs which didn't measure CDRS-R, we will try to convert other depression scales (such as HAMD29 or MADRS30) scores to CDRS-R scores, by using a factor derived from the RCTs that used both scales.<br>As shown in our previous network meta-analysis,13 trial duration varied from 6 weeks to 36 weeks, and the majority of trials employed a treatment duration of 8 weeks. We will try to obtain repeated measures from individual trials if possible. To improve comparability between the included trials, we will prefer the data from 8-week (or the closest to 8-week) time point for efficacy outcomes.<br>(2) Overall tolerability<br>The tolerability of treatment will be the proportion of patients who drop out of the trials early due to side effects at the end of the blinded treatment.<br><br>Secondary outcomes<br>(1) Response rate<br>Response rate will be defined as 50% reduction from baseline to endpoint on CDRS-R (or another standardised rating scale such as HAMD or MADRS).<br>(2) Remission rate<br>Remission rate will be defined as the CDRS scores of less than 28.31<br>(3) Deterioration rate<br>Deterioration represents the depression symptom severity increases after treatment. Deterioration rate will be defined as the proportion of patients whose CDRS-R scores from baseline to endpoint had reliable change index below the cut-off of −1.96.32<br>(4) Overall acceptability<br>The acceptability of treatment will be the proportion of patients who drop out of the trials early for any cause at the end of the blinded treatment.<br>(5) Suicide-related outcomes<br>Suicide-related dichotomous and continuous outcomes will be measured. We will extract the number of participants with suicide-related events (combined suicidal ideation and suicidal behavior) during the acute treatment, as measured on a standardised, validated and reliable rating scale, or reported cases of suicidal ideation and behavior.33 In addition, if data are available, we will also collect data on suicidal ideation as a continuous outcome where a standardised, validated, and reliable rating scale, such as the Suicidal Ideation Questionnaire-Junior High School version (SIQ-JR),34 has been used.	8-9

For peer review only

		(6) Global functioning<br>The outcome of global functioning will be the overall change in validated scales from baseline to endpoint. The commonly used tools of functioning scales included the Children's Global Assessment Scale (CGAS),35 Global Assessment of Functioning (GAF),36 etc.	
Risk of bias in individual studies	14	Two independent review authors will use the Cochrane Collaboration's Risk of bias' tool39 to evaluate the methodological and hence bias risk of eligible studies, and quality assessment will be reported on a study level. The risk of bias will be assessed across seven items, including random sequence generation, allocation concealment, blinding of intervention, blinding of outcome assessment, incomplete outcome data, selective outcome reporting, and other bias, with three level of risk (high, unclear, low). We will rate the quality of study as follows: high risk study (2 or more items rated as high risk of bias); low risk study (5 or more items rated as low risk and no more than one as high risk); unclear risk study (all remaining situations). Any disagreements will be resolved by consensus or consulting the original authors.	12
Data synthesis	15a	All analyses will be performed by intention-to-treat (ITT) analysis. Descriptive statistics will be presented as mean (SD) or median (IQR) for continuous variables and number (percent) for categorical variables.	12
	15b	Individual patient data meta-analyses<br>We will first use the one-stage approach to perform the IPD-MAs, as it offers the highest degree of flexibility for making necessary assumptions, and uses a more exact statistical approach than two-stage approach. We will perform analyses in Stata with the commands mixed (for linear random-effects models), meqrlogit (for logistic models) and ipdforest (for forest plot). To account for between study differences, we will use mixed-effects logistic models for categorical outcomes, and mixed-effects linear regression models for continuous outcomes. Treatment assignment will be introduced as a fixed-effects variable "treatment". As outcomes might vary across studies, we will force the "study" and the interaction term "study*treatment" as random-effects variables into all models. The important clinical and demographic predictors variables (e.g., sex, age, baseline severity score, and treatment duration) will be used as regressors in the models. The heterogeneity of treatment effects across studies will be assessed using the I^2 statistic.	12-13
Meta-bias(es)	16	We will use contour enhanced funnel plot to detect publication bias for study level data (full set of studies meeting inclusion criteria) and patient level data (the set of studies that were included in the IPD-MA), if at least ten studies are available. We will also use Egger's test to quantify the bias, with a *P* value < 0.10 taken to indicate statistical evidence of asymmetry. In order to examine the effects of non-participation of eligible studies, we will conduct a meta-regression analysis with the effect size of primary outcomes (based on study level data) as the dependent variables, and whether or not the patient level data are included as the predictor indicating. The analyses will be conducted in Stata version 14.0.	12
Confidence in cumulative evidence	17	NA.	NA

*** It is strongly recommended that this checklist be read in conjunction with the PRISMA-P Explanation and Elaboration (cite when available) for important clarification on the items. Amendments to a review protocol should be tracked and dated. The copyright for PRISMA-P (including checklist) is held by the PRISMA-P Group and is distributed under a Creative Commons Attribution Licence 4.0.**

From: Shamseer L, Moher D, Clarke M, Ghersi D, Liberati A, Petticrew M, Shekelle P, Stewart L, PRISMA-P Group. Preferred reporting items for systematic review and meta-analysis protocols (PRISMA-P) 2015: elaboration and explanation. BMJ. 2015 Jan 2;349(jan02 1):g7647.

For peer review only

Comparative efficacy and tolerability of new-generation antidepressants for major depressive disorder in children and adolescents: protocol of an individual patient data meta-analysis

Journal:	*BMJ Open*
Manuscript ID	bmjopen-2017-018357.R1
Article Type:	Protocol
Date Submitted by the Author:	13-Oct-2017

Complete List of Authors:	Zhou, Xinyu; The First Affiliated Hospital of Chongqing Medical University, Department of Psychiatry<br>Cipriani, Andrea; University of Oxford, Department of Psychiatry; Warneford Hospital, Oxford Health NHS Foundation Trust<br>Furukawa, Toshi; Kyoto University, Graduate School of Medicine and School of Public Health<br>Cuijpers, Pim; VU University Amsterdam, Department of Clinical Psychology<br>Zhang, Yuqing; The First Affiliated Hospital of Chongqing Medical University, Department of Neurology<br>Hetrick, Sarah; University of Melbourne, Centre for Youth Mental Health<br>Pu, Juncai; The First Affiliated Hospital of Chongqing Medical University, Department of Neurology<br>Yuan, Shuai; The First Affiliated Hospital of Chongqing Medical University, Department of Neurology<br>Del Giovane, Cinzia; University of Modena and Reggio Emilia, Clinical and Diagnostic Medicine and Public Health<br>Xie, Peng; The First Affiliated Hospital of Chongqing Medical University, Department of Neurology; Chongqing Medical University, Institute of Neuroscience and the Collaborative Innovation Center for Brain Science

**Primary Subject Heading**:	Mental health
Secondary Subject Heading:	Mental health
Keywords:	Depression & mood disorders < PSYCHIATRY, child, adolescent, antidepressant, individual patient data meta-analysis, systematic review

Comparative efficacy and tolerability of new-generation antidepressants for major depressive disorder in children and adolescents: protocol of an individual patient data meta-analysis

Xinyu Zhou,[1] Andrea Cipriani,[2,3] Toshi A Furukawa,[4] Pim Cuijpers,[5] Yuqing Zhang,[6] Sarah E. Hetrick,[7] Juncai Pu,[6] Shuai Yuan,[6] Cinzia Del Giovane,[8] Peng Xie[6]

[1] Department of Psychiatry, The First Affiliated Hospital of Chongqing Medical University, Chongqing, China

[2] Department of Psychiatry, University of Oxford, Warneford Hospital, Oxford, UK

[3] Oxford Health NHS Foundation Trust, Warneford Hospital, Oxford, UK

[4] Department of Health Promotion and Human Behavior, Kyoto University Graduate School of Medicine and School of Public Health, Kyoto, Japan

[5] Department of Clinical, Neuro and Developmental Psychology, Amsterdam Public Health research institute, VU University Amsterdam, Amsterdam, The Netherlands

[6] Department of Neurology, The First Affiliated Hospital of Chongqing Medical University, Chongqing, China; Institute of Neuroscience and the Collaborative Innovation Center for Brain Science, Chongqing Medical University, Chongqing, China

[7] Orygen, The National Centre of Excellence in Youth Mental Health, and the Centre of Youth Mental Health, University of Melbourne, Melbourne, Australia

[8] Department of Diagnostic, Clinical and Public Health Medicine, University of Modena and Reggio Emilia, Modena, Italy

Direct correspondence to:

Prof. Peng Xie, Department of Neurology, The First Affiliated Hospital of Chongqing Medical University, 1 Youyi Road, Yuzhong District, Chongqing 400016, China; E-mail: xiepeng973@126.com

Xinyu Zhou, Andrea Cipriani, and Toshi A. Furukawa contributed equally to this work.

Keywords: depression, child, adolescent, antidepressant, individual patient data meta-analysis, systematic review

For peer review only

Word count: 2697.

For peer review only

For peer review only - http://bmjopen.bmj.com/site/about/guidelines.xhtml

ABSTRACT

Introduction: Although previous conventional meta-analyses and network meta-analyses have provided some important findings about pharmacological treatments for children and adolescents with depressive disorders in the past decades, several questions still remain unsolved by the aggregate data from those meta-analyses. Individual participant data meta-analysis (IPD-MA) enables exploration of the impacts of individual characteristics on treatment effects, allowing matching of treatments to specific subgroups of patients. We will perform an IPD-MA to assess the efficacy and tolerability of new-generation antidepressants for major depressive disorder in children and adolescents.

Methods and analysis: We will systematically search for all double-blind randomised controlled trials (RCTs) that have compared any new-generation antidepressant with placebo for the acute treatment of major depressive disorder in children and adolescents, in the following databases: PubMed, EMBASE, the Cochrane Library, PsycINFO, Web of Science, CINAHL, LILACS, and ProQuest Dissertations. We will contact all corresponding authors of included RCTs and ask for their cooperation in this project by providing individual participant data from the original trials. The primary outcomes will include efficacy, measured as the mean change of depression symptoms by CDRS-R scale, and tolerability, measured as the proportion of patients who withdrew from the trials early due to adverse effects. The secondary outcomes will include response rates, remission rates, deterioration rate, all-cause discontinuation, suicidal-related outcomes, as well as global functioning outcome. Using the raw de-identified study data, we will use mixed-effects logistic and linear regression models to perform the IPD-MAs. The risk of bias of included studies will be assessed using the Cochrane risk of bias tool. We will also detect the publication bias and effects of non-participation of eligible studies.

Dissemination: Ethical approval is not required given that informed consent has already been obtained from the patients by the trial investigators before the included trials were conducted. This study may have considerable implications for practice and help improve patient care.

Protocol registration: PROSPERO CRD42016051657

For peer review only

For peer review only - http://bmjopen.bmj.com/site/about/guidelines.xhtml

Strengths and limitations of this study

Strengths:

1. This is the first individual participant data meta-analysis (IPD-MA) comparing the efficacy and tolerability of new-generation antidepressants for major depressive disorder in children and adolescents.

2. The study will use individual patient data that can take into account within-study and between-study differences, yield more reliable estimates of treatment effects than meta-analysis of aggregate data.

3. Individual patient data meta-analysis can provide insight into the patient groups most likely to benefit from new-generation antidepressants and the most effective kinds of antidepressants.

Limitations:

1. It is difficult to ensure all trials were identified, because not all trials are registered, especially for these old trials.

2. The another difficulty of this study will be collecting the patient-level information from all eligible trials, for some of the original investigators may not be willing or able to share the data. For example for the fluoxetine trials, European medicines agency (EMA) did not have them and Medicines and healthcare products regulatory agency (MHRA) were to have saved the records but they could only find three placebo controlled double blind RCTs, the other records had been destroyed as per their policy of older reports.

3. We found that different CSRs depending on the company and the time of the study varied significantly with respect to quality. Therefore, this definitely data would rely on getting access to databases etc for complete data.

For peer review only - http://bmjopen.bmj.com/site/about/guidelines.xhtml

Background

Major depressive disorder (MDD) is a commonly occurring serious mental disorder, accounting for a large portion of the global burden of disease. The overall prevalence rate of depressive disorder is about 3% in children and 6% in adolescents.[1] Depressive disorder in youth is often associated with high rates of comorbid mental disorders, functional impairment, and suicide.[2-5] For young people aged 10–19 years, depressive disorders are the leading cause of health-related burden, accounting for 6–10% of the disability-adjusted life-years.[6] Early-onset depression is an important predictor of the recurrence of depressive disorders. In a naturalistic follow up study, up to 55% pediatric patients who recovered from the first episode of MDD, had a second episode within 5 years and rose to 72% within 15 years.[7]

In the past 20 years, several new-generation antidepressants have been found to be effective in the treatment of adult MDD.[8,9] However, whether to use antidepressants in children and adolescents are still matters of controversy, mainly due to concerns about efficacy and potentially increased risk of treatment-emergent suicide in those young patients.[10,11] In 2004, some worrying interpretations from a conventional meta-analysis were shown: published data suggested a favorable risk benefit profile for some selective serotonin reuptake inhibitors (SSRIs); however, addition of unpublished data indicated that risks could outweigh the benefits of these drugs (except fluoxetine) for the treatment of depression in children and young people.[12] Recently, our published network meta-analysis showed that most currently available antidepressants do not seem to offer a clear advantage over placebo for depression in children and adolescents, and fluoxetine is probably the best option to consider when a pharmacological treatment is indicated.[13] Nevertheless, several questions still remain unsolved by the aggregate data from conventional and network meta-analyses. First, the effect sizes of some antidepressants in previous meta-analyses had large confidence/credible interval with its upper limit close to the point of no difference, which raises the question of whether this estimate is robust enough to inform clinical practice.[14] Second, most studies included both children and adolescents, but they did not separately report the data of different age groups. Thus, it remains unclear whether the antidepressants are efficacious across the diverse populations included. Third, there was strict range of baseline severity scores included in these previous meta-analyses. For example, in our previous NMA analysis, most studies focused on samples with moderate to severe depressive severity, with few trials of those with mild to moderate, or very severe range. Therefore, whether the antidepressants have similar efficacy for mildly or severely depressed patients is another important question that remains. Fourth, RCTs evaluating antidepressant treatments in children and adolescents seldom report the number of patients who deteriorated during treatment, thus it is not possible to investigate mean deterioration effects found

For peer review only - http://bmjopen.bmj.com/site/about/guidelines.xhtml

in randomized trials and its moderators using conventional and network meta-analytical approaches.

Individual participant data meta-analysis (IPD-MA) is an increasingly popular approach for synthesizing and investigating treatment effect estimates. IPD-MA has many statistical and clinical advantages over meta-analyses of aggregate data. For example, clinical heterogeneity can be reduced by controlling for patient-level covariates in IPD-MA,[15,16] which offers the potential to explore additional, more thorough, and potentially more appropriate analyses compared to those possible with aggregate data.[17] IPD-MA also provides unique opportunities to identify underlying individual characteristics as prognostic factors or negative effects across several studies.[18] Therefore, we will perform an IPD-MA to assess the efficacy and tolerability of new-generation antidepressants for major depressive disorder in children and adolescents.

Methods

Criteria for included studies

Types of studies

Studies included in this IPD-MA will be double-blind randomised controlled trials (RCTs), including studies with cluster or cross-over designs. Given possible carry-over effects, we will only consider data from the first study period in cross-over trials. We will exclude trials employing inappropriate randomisation strategies, such as quasi-randomised designs.

Types of participants

Studies will be included in the IPD-MA if they aim at (1) children and adolescents aged between 6–18 years when initially enrolled in the studies, (2) with primary diagnosis of major depressive disorder according to standard diagnostic criterion, such as the Diagnostic and Statistical Manual of Mental Disorders (DSM),[19-23] or the International Classification of Diseases (ICD).[24,25] Studies will be excluded if they included patients with bipolar depression, or treatment-resistant depression, while patients with comorbid general psychiatric disorders, such as anxiety disorder, will not be excluded.

Types of interventions

We will include all RCTs comparing any new-generation antidepressant with placebo during the acute treatment phase of depression in children and adolescents. The following new-generation antidepressants using prescribed oral and therapeutic dose range will be included.[8,13,26]

1. Selective serotonin reuptake inhibitors (SSRIs), e.g., fluoxetine, fluvoxamine, paroxetine, sertraline, citalopram, and escitalopram.

2. Serotonin and norepinephrine reuptake inhibitors (SNRIs), e.g., duloxetine, venlafaxine, desvenlafaxine, milnacipran, levomilnacipran.

For peer review only - http://bmjopen.bmj.com/site/about/guidelines.xhtml

3. Other antidepressants, e.g., mirtazapine, mianserin, nefazodone, trazodone, vortioxetine, vilazodone, bupropion, reboxetine, and agomelatine.

We will only include RCTs with a minimum of 4-week treatment duration, because the onset of benefit for most antidepressants often takes at least 4 weeks.[27] We will exclude trials designed as maintenance treatment or relapse prevention, unless outcome data from the acute phase can be accessed separately. Combination studies and augmentation studies (e.g. combined with different antidepressant or psychotherapy) will also be excluded.

Types of outcome measures

Primary outcomes

(1) Overall efficacy

The primary outcome of efficacy will be the overall change in depressive symptoms, as measured using Children's Depression Rating Scale Revised (CDRS-R[28]) from baseline to endpoint. For RCTs which didn't measure CDRS-R, we will try to convert other depression scales (such as HAMD[29] or MADRS[30]) scores to CDRS-R scores, by using a factor derived from the RCTs that used both scales.

As shown in our previous network meta-analysis,[13] trial duration varied from 6 weeks to 36 weeks, and the majority of trials employed a treatment duration of 8 weeks. We will try to obtain repeated measures from individual trials if possible. To improve comparability between the included trials, we will prefer the data from 8-week (or the closest to 8-week) time point for efficacy outcomes.

(2) Overall tolerability

The tolerability of treatment will be the proportion of patients who drop out of the trials early due to side effects at the end of the blinded treatment.

Secondary outcomes

(1) Response rate

Response rate will be defined as 50% reduction from baseline to endpoint on CDRS-R (or another standardised rating scale such as HAMD or MADRS).

(2) Remission rate

Remission rate will be defined as the CDRS scores of less than 28.[31]

(3) Deterioration rate

Deterioration represents the depression symptom severity increases after treatment. Deterioration rate will be defined as the proportion of patients whose CDRS-R scores from baseline to endpoint had reliable change index below the cut-off of −1.96.[32]

(4) Overall acceptability

The acceptability of treatment will be the proportion of patients who drop out of the trials early for any cause at the end of the blinded treatment.

(5) Suicide-related outcomes

For peer review only

For peer review only - http://bmjopen.bmj.com/site/about/guidelines.xhtml

Suicide-related dichotomous and continuous outcomes will be measured. We will extract the number of participants with suicide-related events (combined suicidal ideation and suicidal behavior) during the acute treatment, as measured on a standardised, validated and reliable rating scale, or reported cases of suicidal ideation and behavior.[33] In addition, if data are available, we will also collect data on suicidal ideation as a continuous outcome where a standardised, validated, and reliable rating scale, such as the Suicidal Ideation Questionnaire-Junior High School version (SIQ-JR),[34] has been used.

(6) Global functioning

The outcome of global functioning will be the overall change in validated scales from baseline to endpoint. The commonly used tools of functioning scales included the Children's Global Assessment Scale (CGAS),[35] Global Assessment of Functioning (GAF),[36] etc.

(7) Aggressive behaviour

The outcome of aggressive behaviour will be the proportion of cases who reported the aggressive behaviour, such as hostility and assault, during the acute treatment. [37,38]

Data Sources and Search strategy

We will first include the RCTs identified by the criteria used in our previous work.[13,39] Then we will update the extensive searching to bring it up to date. Briefly, we will identify any published and unpublished RCTs, in any language, from electronic systematic searches of PubMed, EMBASE, the Cochrane Library, PsycINFO, Web of Science, LILACS, CINAHL, and ProQuest Dissertations. Electronic databases will be searched with free words and Medical Subject Headings (MeSH) terms using the following strategy: [depress* or dysthymi* or mood disorder* or affective disorder*], combined with [adolesc* or child* or boy* or girl* or juvenil* or minors or paediatri* or pediatri* or pubescen* or school* or student* or teen* or young or youth*], and combined with a list of antidepressants, including [selective serotonin reuptake inhibitors or SSRI or fluoxetine or fluvoxamine or paroxetine or sertraline or citalopram or escitalopram or serotonin and norepinephrine reuptake inhibitors or SNRI or duloxetine or venlafaxine or desvenlafaxine or milnacipran or levomilnacipran or mirtazapine or mianserin or nefazodone or trazodone or vortioxetine or vilazodone or bupropion or reboxetine or agomelatine]. In addition, we will also identify additional trials and unpublished data by searching: (1) international trials registries, mainly including of ClinicalTrials.gov, and World Health Organization International Clinical Trials Registry Platform (ICTRP); (2) USA Food and Drug Administration (FDA) reports; (3) the International Prospective Register of Systematic Reviews (PROSPERO); (4) websites of main manufactures, e.g., GlaxoSmithKline, Lilly, Organon, Forest Pharmaceuticals, Bristol-Myers Squibb; (5) manual hand search of key journals and conference proceedings, e.g., *J Child Adolesc Psychopharmacol, J Am Acad Child Adolesc Psychiatry, Child Adolesc Psychiatry Ment Health, Psychopharmacol Bull, Arch Gen Psychiatry, Am J Psychiatry, Eur Psychiatry, Depress Anxiety*. Additional relevant RCTs will be obtained by hand-searching reference lists of included studies and relevant reviews. We will also contact corresponding authors of included RCTs, manufactures, FDA, and other possible institutions for unpublished trials.

For peer review only - http://bmjopen.bmj.com/site/about/guidelines.xhtml

For peer review only

Study selection and data extraction

Selection of trials

We will first manually remove duplicates of initial search results. Then two experienced reviewers will independently screen titles and abstracts from the retrieved results for possible candidates. We will exclude the trials in which both reviewers judge they do not meet eligibility criteria. Full texts of all remaining papers will be retrieved, and two reviewers will independently examine whether to include them by the same eligibility criteria. Any difference of opinion, for each step, between the reviewers will be resolved through discussion with another member of the reviewing team, or by contacting the authors of the trials for clarification. The selection process of retrieved studies and the reasons for exclusion of trials (e.g. ineligible populations, not randomized trials) will be shown in a flow chart.

Data collection

From the included RCTs, two reviewers will independently extract the trial level information using standardized data collection forms, including trial characteristics, patient characteristics, intervention details, and any other information relevant to this review.

We will contact all corresponding authors or sponsor pharmaceutical companies of included RCTs and ask for their cooperation in this project. The corresponding authors' contact information will be abstracted from the papers, online research profiles (e.g., Google Scholar), or other available ways. Specifically, we will (1) send emails to the authors explaining the study purpose, and invite them to cooperate in this project; (2) send reminder emails 4 and 6 weeks later if no response; (3) contact the corresponding authors by phone or possible personal contacts. We will also report on the process of interaction with the sponsor companies, as applicable.

Trial level information and individual participant data to be obtained from the original authors are shown in Table 1, respectively. The raw data can be provided in any convenient manner (such as by email) in common types of electronic format, such as Excel, SPSS, Stata, etc. All obtained data will be converted to a uniform format, and saved on a secure server at the Chongqing Medical University. The data set will not contain any personal identifier of patients, such as names or phone numbers. Only authorised members of the research team will be allowed to access the data set.

For peer review only

Data checking

We will check for data-entry mistakes and consistency, and reanalyse the data within each study according to the original statistical methodology, the results will be compared with the published summary results. Any error will be resolved by discussion with the original investigators, and data corrections will be made if necessary.

Missing data

Handling of missing data will depend on the proportion of missing data in the full dataset. In general, we

For peer review only - http://bmjopen.bmj.com/site/about/guidelines.xhtml

will prefer to manage missing data for both patient characteristics and outcomes through multiple imputation methods, such as multiple imputation (MI) and mixed-effects model repeated measures (MMRM), because multiple imputation techniques with a missing at random assumption tends to yield more unbiased results than single imputation methods.[40] Missing data will be imputed using the command *mi impute mvn* in Stata version 14.0. However, if we obtain repeated measures from individual trials, we will use MMRM approach.

Risk of bias assessment and quality of study

Two independent review authors will use the Cochrane Collaboration's Risk of bias' tool[41] to evaluate the methodological and hence bias risk of eligible studies, and quality assessment will be reported on a study level. The risk of bias will be assessed across seven items, including random sequence generation, allocation concealment, blinding of intervention, blinding of outcome assessment, incomplete outcome data, selective outcome reporting, and other bias (for instance, conflicts of interests) with three level of risk (high, unclear, low). We will rate the quality of study as follows: high risk study (2 or more items rated as high risk of bias); low risk study (5 or more items rated as low risk and no more than one as high risk); unclear risk study (all remaining situations). Any disagreements will be resolved by consensus or consulting the original authors.

Publication bias and effects of non-participation of eligible studies

We will use contour enhanced funnel plot to detect publication bias for study level data (full set of studies meeting inclusion criteria) and patient level data (the set of studies that were included in the IPD-MA), if at least ten studies are available.[42] We will also use Egger's test to quantify the bias, with a P value < 0.10 taken to indicate statistical evidence of asymmetry.[43] In order to examine the effects of non-participation of eligible studies, we will conduct a meta-regression analysis with the effect size of primary outcomes (based on study level data) as the dependent variables, and whether or not the patient level data are included as the predictor indicating. The analyses will be conducted in Stata version 14.0.

Statistical analysis

All analyses will be performed by intention-to-treat (ITT) analysis. Descriptive statistics will be presented as mean (SD) or median (IQR) for continuous variables and number (percent) for categorical variables.

Individual patient data meta-analyses

We will first use the one-stage approach to perform the IPD-MAs, as it offers the highest degree of flexibility for making necessary assumptions,[44] and uses a more exact statistical approach than two-stage approach.[45] We will perform analyses in Stata with the commands *mixed* (for linear random-effects models), *meqrlogit* (for logistic models) and *ipdforest* (for forest plot).[46] To account for between study differences, we will use mixed-effects logistic models for categorical outcomes, and mixed-effects linear regression models for continuous outcomes. Treatment assignment will be introduced as a fixed-effects variable "treatment". As outcomes might vary across studies, we will force the "study" and the interaction term "study*treatment" as

For peer review only - http://bmjopen.bmj.com/site/about/guidelines.xhtml

random-effects variables into all models. The important clinical and demographic predictors variables (e.g., sex[47], age[48], baseline severity score[49], and treatment duration) will be used as regressors in the models. The heterogeneity of treatment effects across studies will be assessed using the I^2 statistic.[50] Finally, we will carry out the following sensitivity analyses of the primary outcomes: (i) excluding trials with a follow up longer than 12 weeks and (ii) excluding studies where HAMD and MADRS scores were mapped onto CDRS-R.

Ethics and dissemination

This protocol is registered in the International Prospective Register of Systematic Reviews (PROSPERO) at the National Health Service Centre for Reviews and Dissemination at the University of York (Registration number: CRD42016051657).No ethics review is required for this IPD meta-analysis, since informed consent has already been obtained from the patients by the trial investigators before the trial was conducted. We will publish the results in a peer-reviewed journal.

For peer review only

For peer review only - http://bmjopen.bmj.com/site/about/guidelines.xhtml

Contributors

PX and XZ conceived the study and wrote the first draft of the protocol, and will assist with the data extraction and analysis, and draft the results and discussion sections. AC, TAF, PC, SEH, CDG assisted in protocol design and revision, and will help draft the final manuscript. XZ, YZ, JP and SY participated in the search strategy development, will carry out most of the data collection. CDG, AC and TAF participated in the design of data synthesis and analysis, and will carry out the statistical analyses. All the authors have approved the publication of the protocol.

Funding This work is supported by the National Basic Research Program of China (973 Program) (Grant No. 2009CB918300). The funders had no role in the protocol design; the writing of the protocol; or the decision to submit the protocol for publication.

Competing interests AC reports personal fees from Accord Healthcare as an expert witness for a patent issue about quetiapine extended release. TAF has received lecture fees from Eli Lilly, Janssen, Meiji, MSD, Otsuka, Pfizer and Tanabe-Mitsubishi, and consultancy fees from Sekisui Chemicals and Takeda Science Foundation. He has received royalties from Igaku-Shoin and Nihon Bunka Kagaku-sha publishers. He has received research support from Mochida and Tanabe-Mitsubishi. SEH is an Editor of the Cochrane Common Mental Disorders Group, an author of the Cochrane systematic review of newer generation antidepressants for depression in children and adolescents, and an author (senior) on the Cochrane review of psychological, pharmacological and their combination for child/adoles depression. XZ, PC, YZ, JP, SY, CDG, and PX declare no competing interests.

Acknowledgments Andrea Cipriani is supported by the National Institute for Health Research Oxford Cognitive Health Clinical Research Facility.

Provenance and peer review Not commissioned; externally peer-reviewed.

Open Access This is an Open Access article distributed in accordance with the Creative Commons Attribution- Non Commercial (CC BY-NC 4.0) license, which permits others to distribute, remix, adapt, build upon this work non-commercially, and license their derivative works on different terms, provided the original work is properly cited and the use is non-commercial. See: http://creativecommons.org/licenses/by-nc/4.0/

For peer review only - http://bmjopen.bmj.com/site/about/guidelines.xhtml

References

1. Jane Costello E, Erkanli A, Angold A. Is there an epidemic of child or adolescent depression? *J Child Psychol Psychiatry* 2006;47(12):1263-71.
2. Hawton K, Saunders KE, O'Connor RC. Self-harm and suicide in adolescents. *Lancet* 2012;379(9834):2373-82.
3. Rohde P, Lewinsohn PM, Klein DN, *et al*. Key characteristics of major depressive disorder occurring in childhood, adolescence, emerging adulthood, adulthood. *Clin Psychol Sci* 2013;1(1):41-53.
4. Jaffee SR, Moffitt TE, Caspi A, *et al*. Differences in early childhood risk factors for juvenile-onset and adult-onset depression. *Arch Gen Psychiatry* 2002;59(3):215-22.
5. Siu AL, U.S. Preventive Services Task Force. Screening for depression in children and adolescents: U.S. Preventive Services Task Force recommendation statement. *Ann Intern Med* 2016; 164(5):360-6.
6. Gore FM, Bloem PJ, Patton GC, *et al*. Global burden of disease in young people aged 10-24 years: a systematic analysis. *Lancet* 2011;377(9783):2093-102.
7. Kovacs M, Obrosky S, George C. The course of major depressive disorder from childhood to young adulthood: recovery and recurrence in a longitudinal observational study. *J Affect Disord* 2016;203:374-81.
8. Cipriani A, Furukawa TA, Salanti G, *et al*. Comparative efficacy and acceptability of 12 new-generation antidepressants: a multiple-treatments meta-analysis. *Lancet* 2009;373(9665):746-58.
9. Kennedy SH, Lam RW, Mcintyre RS, *et al*. Canadian Network for Mood and Anxiety Treatments (CANMAT) 2016 clinical guidelines for the management of adults with major depressive disorder: section 3. Pharmacological treatments. *Can J Psychiatry* 2016;61(9):540-60.
10. Bridge JA, Iyengar S, Salary CB, *et al*. Clinical response and risk for reported suicidal ideation and suicide attempts in pediatric antidepressant treatment: a meta-analysis of randomized controlled trials. *JAMA* 2007;297(15):1683-96.
11. U.S. Food and Drug Administration (FDA). Suicidality in children and adolescents being treated with antidepressant medications. www.fda.gov/drugs/drugsafety/postmarketdrugsafetyinformationforpatientsandproviders/ucm161679.htm (accessed 21 Jun 2017).
12. Whittington CJ, Kendall T, Fonagy P, *et al*. Selective serotonin reuptake inhibitors in childhood depression: systematic review of published versus unpublished data. *Lancet* 2004;363(9418):1341-5.
13. Cipriani A, Zhou X, Del Giovane C, *et al*. Comparative efficacy and tolerability of antidepressants for major depressive disorder in children and adolescents: a network meta-analysis. *Lancet* 2016;388(10047):881-90.
14. Salanti G, Del Giovane C, Chaimani A, *et al*. Evaluating the quality of evidence from a network meta-analysis. *PLoS One* 2014;9(7):e99682.
15. Riley RD, Lambert PC, Abo-Zaid G. Meta-analysis of individual participant data: rationale, conduct, and reporting. *BMJ* 2010;340(7745):521-25.
16. Debray TP, Moons KG, Ahmed I, *et al*. A framework for developing, implementing, and evaluating

For peer review only - http://bmjopen.bmj.com/site/about/guidelines.xhtml

clinical prediction models in an individual participant data meta-analysis. *Stat Med* 2013;32(18):3158-80.

17. Tudur Smith C, Marcucci M, Nolan SJ, *et al*. Individual participant data meta-analyses compared with meta-analyses based on aggregate data. *Cochrane Database Syst Rev* 2016, 9:MR000007.
18. Debray TP, Riley RD, Rovers MM, *et al*. Individual participant data (IPD) meta-analyses of diagnostic and prognostic modeling studies: guidance on their use. *PLoS Med* 2015;12(10):e1001886.
19. American Psychiatric Association. *Diagnostic and statistical manual of mental disorders (DSM-III)*. 3rd Edition. Washington DC: American Psychiatric Association, 1980.
20. American Psychiatric Association. *Diagnostic and statistical manual of mental disorders (DSM-III-R)*. 3rd Edition. Washington DC: American Psychiatric Association, 1987.
21. American Psychiatric Association. *Diagnostic and statistical manual of mental disorders (DSM-IV)*. 4th Edition. Washington DC: American Psychiatric Association, 1994.
22. American Psychiatric Association. *Diagnostic and statistical manual of mental disorders (DSM-IV-TR)*. 4th Edition. Washington DC: American Psychiatric Association, 2000.
23. American Psychiatric Association. *Diagnostic and statistical manual of mental disorders (DSM-5)*. 5th Edition. Arlington, VA: American Psychiatric Publishing, 2013.
24. World Health Organization (WHO). *The Ninth Revision of the International Classification of Diseases and Related Health Problems (ICD-9)*. Geneva: World Health Organization, 1978.
25. World Health Organization. *The ICD-10 classification of mental and behavioural disorders: clinical description and diagnostic guidelines*. Geneva: World Health Organization, 1992.
26. Hetrick SE, McKenzie JE, Cox GR, *et al*. Newer generation antidepressants for depressive disorders in children and adolescents. *Cochrane Database Syst Rev* 2012;11(11):CD004851.
27. American Psychiatric Association. *Guideline watch: practice guideline for the treatment of patients with major depressive disorder*. 3nd edn. http://psychiatryonline.org/pb/assets/raw/sitewide/practice_guidelines/guidelines/mdd.pdf (accessed 21 Jun 2017).
28. Poznanski EO, Mokros HB. *Children's depression rating scale, revised (CDRS-R): manual.* Los Angeles, CA: Western Psychological Services, 1996.
29. Hamilton M. A rating scale for depression. *J Neurol Neurosurg Psychiatry* 1960;23:56-62.
30. Montgomery SA, Asberg M. A new depression scale designed to be sensitive to change. *Br J Psychiatry* 1979;134:382-9.
31. Emslie GJ, Rush AJ, Weinberg WA, *et al*. A double-blind, randomized, placebo-controlled trial of fluoxetine in children and adolescents with depression. *Arch Gen Psychiatry* 1997; 54(11):1031-7.
32. Jacobson N, Truax P. Clinical significance: a statistical approach to defining meaningful change in psychotherapy research. *J Consult Clin Psychol* 1991;59(1):12-9.
33. U.S. Food and Drug Administration (FDA). *Guidance for industry: suicidal ideation and behavior: prospective assessment of occurrence in clinical trials. Draft guidance.* 1st revision. http://www.fda.gov/drugs/guidancecomplianceregulatoryinformation/guidances/ucm315156.htm

For peer review only

For peer review only - http://bmjopen.bmj.com/site/about/guidelines.xhtml

(accessed 21 Jun 2017).

34. Reynolds WM. *Professional Manual for the Suicidal Ideation Questionnaire*. Odessa, FL: Psychological Assessment Resources, 1988.
35. Shaffer D, Gould MS, Brasic J, *et al*. A children's global assessment scale (CGAS). *Arch Gen Psychiatry* 1983;40(11):1228-31.
36. Endicott J, Spitzer RL, Fleiss JL, *et al*. The global assessment scale: a procedure for measuring overall severity of psychiatric disturbance. *Arch Gen Psychiatry* 1976;33(6):766-71.
37. Sharma T, Guski LS, Freund N, Gøtzsche PC. Suicidality and aggression during antidepressant treatment: systematic review and meta-analyses based on clinical study reports. BMJ. 2016;352:i65.
38. Moore TJ, Glenmullen J, Furberg CD. Prescription drugs associated with reports of violence towards others. PLoS One 2010;5: e15337.
39. Zhou X, Qin B, Whittington C, *et al*. Comparative efficacy and tolerability of first-generation and newer-generation antidepressant medications for depressive disorders in children and adolescents: study protocol for a systematic review and network meta-analysis. *BMJ Open* 2015;5(9):e007768.
40. DondersAR, van der Heijden GJ, Stijnen T, *et al*. Review: a gentle introduction to imputation of missing values. *J Clin Epidemiol* 2006;59(10):1087-91.
41. Higgins JPT, Green S. *Cochrane handbook for systematic reviews of interventions version 5.1. 0* [updated March 2011]. The Cochrane Collaboration, 2011. http://handbook.cochrane.org/ (accessed 21 Jun 2017).
42. Sterne JA, Sutton AJ, Ioannidis JP, *et al*. Recommendations for examining and interpreting funnel plot asymmetry in meta-analyses of randomised controlled trials. *BMJ* 2011;343:d4002.
43. Egger M, Davey Smith G, Schneider M, *et al*. Bias in meta-analysis detected by a simple, graphical test. *BMJ* 1997;315(7129):629-34.
44. Debray TP, Moons KG, van Valkenhoef G, *et al*. Get real in individual participant data (IPD) meta-analysis: a review of the methodology. *Res Synth Methods* 2015;6(4):293-309.
45. Debray TP, Moons KG, Abo-Zaid GM, *et al*. Individual participant data meta-analysis for a binary outcome: one-stage or two-stage? *PLoS One* 2013;8(4):e60650.
46. Kontopantelis E, Reeves D. A short guide and a forest plot command (ipdforest) for one-stage meta-analysis. *Stata J* 2013;13(3):574-87.
47. Sotsky SM, Glass DR, Shea MT, *et al*. Patient predictors of response to psychotherapy and pharmacotherapy: findings in the NIMH Treatment of Depression Collaborative Research Program. *Am J Psychiatry* 1991;148(8):997-1008.
48. Kowatch RA, Carmody TJ, Emslie GJ, *et al*. Prediction of response to fluoxetine and placebo in children and adolescents with major depression: a hypothesis generating study. *J Affect Disord* 1999; 54(3):269-76.
49. Curry J, Rohde P, Simons A, *et al*. Predictors and moderators of acute outcome in the Treatment for Adolescents With Depression Study (TADS). *J Am Acad Child Adolesc Psychiatry 2006*, 45(12):1427-39.
50. Higgins JP, Thompson SG, Deeks JJ, *et al*. Measuring inconsistency in meta-analyses. *BMJ* 2003;327(7414):557-60.

For peer review only

TABLES

Table 1. Data items to be requested for individual participant data meta-analysis

Trial level information	Demographic and baseline characteristics	Therapeutic process	Outcomes
1. Study protocol<br>2. Clinical study report (if available);<br>3. List of publications<br>4. Setting (such as primary care, hospitals, clinics)<br>5. Information about the risk of bias (sequence generation, allocation of concealment, masking, and ITT analysis)<br>6. Any other information relevant to this review	1. Unique identification number for anonymity<br>2. Date of randomization<br>3. Sex (Male, female)<br>4. Race (White/Caucasian, African/African-American, Asian, Multiracial, Other)<br>5. Body mass index (BMI), kg/m^2<br>6. Height, cm<br>7. Weight, kg<br>8. Age, year<br>9. Age at onset, year<br>10. Length of illness, month<br>11. Number of MDD episodes<br>12. Duration of current episode, month<br>13. Baseline depression symptom score<br>14. Baseline quality of life and functioning score<br>15. Previous and/or ongoing secondary psychiatric disorder (anxiety disorder, externalizing disorder (ADHD, conduct disorder, oppositional defiant disorder), psychotic disorder, substance-related disorder)<br>16. Family history of MDD<br>17. Household (Two parents, other)<br>18. Number of siblings<br>19. Life and social history<br>20. Previous suicide attempt	1. Treatment (antidepressant, placebo)<br>2. Dose range<br>3. Total actual drug exposure<br>4. Treatment duration<br>5. Co-prescriptions other than antidepressant<br>6. Prior treatments (no therapy, psychotherapy, pharmacotherapy, both psychotherapy and pharmacotherapy)	1. Depression symptom scores at each evaluation (scale, time point)<br>2. Quality of life and functioning scores at each evaluation (scale, time point)<br>3. Study discontinuation and reason (dropout before starting the treatment, lack of efficacy, adverse events, others)<br>4. Adverse events<br>5. Serious adverse events (SAEs)<br>6. Suicide-related event

For peer review only - http://bmjopen.bmj.com/site/about/guidelines.xhtml

For peer review only

PRISMA-P (Preferred Reporting Items for Systematic review and Meta-Analysis Protocols) 2015 checklist: recommended items to address in a systematic review protocol*

Section and topic	Item No	Checklist item	Reported on Page #
**ADMINISTRATIVE INFORMATION**			
Title:			
Identification	1a	Comparative efficacy and tolerability of new-generation antidepressants for major depressive disorder in children and adolescents: protocol of an individual patient data meta-analysis	1
Update	1b	None	
Registration	2	PROSPERO CRD42016051657	3
Authors:			
Contact	3a	Xinyu Zhou (Department of Psychiatry, The First Affiliated Hospital of Chongqing Medical University, Chongqing, China)<br>Andrea Cipriani (Department of Psychiatry, University of Oxford, Warneford Hospital, Oxford, UK; Oxford Health NHS Foundation Trust, Warneford Hospital, Oxford, UK)<br>Toshi A. Furukawa (Department of Health Promotion and Human Behavior, Kyoto University Graduate School of Medicine and School of Public Health, Kyoto, Japan)<br>Pim Cuijpers (Department of Clinical, Neuro and Developmental Psychology, Amsterdam Public Health research institute, VU University Amsterdam, Amsterdam, The Netherlands)<br>Yuqing Zhang, Juncai Pu, Shuai Yuan, Peng Xie (Department of Neurology, The First Affiliated Hospital of Chongqing Medical University, Chongqing, China; Institute of Neuroscience and the Collaborative Innovation Center for Brain Science, Chongqing Medical University, Chongqing, China)<br>Sarah E. Hetrick (Orygen, The National Centre of Excellence in Youth Mental Health, and the Centre of Youth Mental Health, University of Melbourne, Melbourne, Australia)<br>Cinzia Del Giovane (Department of Diagnostic, Clinical and Public Health Medicine, University of Modena and Reggio Emilia, Modena, Italy)<br><br>Corresponding author :Peng Xie, Department of Neurology, The First Affiliated Hospital of Chongqing Medical University, 1 Youyi Road, Yuzhong District, Chongqing 400016, China; E-mail: xiepeng973@126.com	1
Contributions	3b	PX and XZ conceived the study and wrote the first draft of the protocol, and will assist with the data extraction and analysis, and draft the results and discussion sections. AC, TAF, PC, SEH, CDG assisted in protocol design and revision, and will help draft the final manuscript. XZ, YZ, JP and SY participated in the search strategy development, will carry out most of the data collection. CDG, AC and TAF participated in the design of data synthesis and analysis, and will carry out the statistical analyses. All the authors have approved the publication of the protocol.	12
Amendments	4	None	

For peer review only

Support:			
Sources	5a	None	
Sponsor	5b	This work is supported by the National Basic Research Program of China (973 Program) (Grant No. 2009CB918300).	12
Role of sponsor or funder	5c	The funders had no role in the protocol design; the writing of the protocol; or the decision to submit the protocol for publication.	
**INTRODUCTION**			
Rationale	6	Depressive disorder in children and adolescents is a major public health problem. The course of depressive disorder in young people is often characterised by protracted episodes, frequent recurrence, and comorbid psychiatric disorders. In the past 20 years, several new-generation antidepressants have been found to be effective in the treatment of adult MDD. However, whether to use antidepressants in children and adolescents are still matters of controversy, mainly due to concerns about efficacy and potentially increased risk of treatment-emergent suicide in those young patients. Recently, our published network meta-analysis showed that most currently available antidepressants do not seem to offer a clear advantage over placebo for depression in children and adolescents, and fluoxetine is probably the best option to consider when a pharmacological treatment is indicated.13 Nevertheless, several questions still remain unsolved by the aggregate data from conventional and network meta-analyses. Individual participant data meta-analysis (IPD-MA) is an increasingly popular approach for synthesizing and investigating treatment effect estimates. IPD-MA has many statistical and clinical advantages over meta-analyses of aggregate data.	5-6
Objectives	7	Therefore, we will perform an IPD-MA to assess the efficacy and tolerability of new-generation antidepressants for major depressive disorder in children and adolescents.	6
**METHODS**			
Eligibility criteria	8	**Types of participants**<br>Studies will be included in the IPD-MA if they aim at (1) children and adolescents aged between 6–18 years when initially enrolled in the studies, (2) with primary diagnosis of major depressive disorder according to standard diagnostic criterion, such as the Diagnostic and Statistical Manual of Mental Disorders (DSM), or the International Classification of Diseases (ICD). Studies will be excluded if they included patients with bipolar depression, or treatment-resistant depression, while patients with comorbid general psychiatric disorders, such as anxiety disorder, will not be excluded.<br><br>**Types of studies**<br>Studies included in this IPD-MA will be double-blind randomised controlled trials (RCTs), including studies with cluster or cross-over designs. Given possible carry-over effects, we will only consider data from the first study period in cross-over trials. We will exclude trials employing inappropriate randomisation strategies, such as quasi-randomised designs.<br><br>**Types of interventions**<br>We will include all RCTs comparing any new-generation antidepressant with placebo during the acute treatment phase of depression in children and adolescents. The following new-generation antidepressants using prescribed oral and therapeutic dose range will be included.	6-7

For peer review only

		1. Selective serotonin reuptake inhibitors (SSRIs), e.g., fluoxetine, fluvoxamine, paroxetine, sertraline, citalopram, and escitalopram.<br>2. Serotonin and norepinephrine reuptake inhibitors (SNRIs), e.g., duloxetine, venlafaxine, desvenlafaxine, milnacipran, levomilnacipran.<br>3. Other antidepressants, e.g., mirtazapine, mianserin, nefazodone, trazodone, vortioxetine, vilazodone, bupropion, reboxetine, and agomelatine.<br>We will only include RCTs with a minimum of 4-week treatment duration, because the onset of benefit for most antidepressants often takes at least 4 weeks. We will exclude trials designed as maintenance treatment or relapse prevention, unless outcome data from the acute phase can be accessed separately. Combination studies and augmentation studies (e.g. combined with different antidepressant or psychotherapy) will also be excluded.	
Information sources	9	We will first include the RCTs identified by the criteria used in our previous work. Then we will update the extensive searching to bring it up to date. Briefly, we will identify any published and unpublished RCTs, in any language, from electronic systematic searches of PubMed, EMBASE, the Cochrane Library, PsycINFO, Web of Science, LILACS, CINAHL, and ProQuest Dissertations. In addition, we will also identify additional trials and unpublished data by searching: (1) international trials registries, mainly including of ClinicalTrials.gov, and World Health Organization International Clinical Trials Registry Platform (ICTRP); (2) USA Food and Drug Administration (FDA) reports; (3) websites of main manufactures, e.g., GlaxoSmithKline, Lilly, Organon, Forest Pharmaceuticals, Bristol-Myers Squibb; (3) manual hand search of key journals and conference proceedings, e.g., J Child Adolesc Psychopharmacol, J Am Acad Child Adolesc Psychiatry, Child Adolesc Psychiatry Ment Health, Psychopharmacol Bull, Arch Gen Psychiatry, Am J Psychiatry, Eur Psychiatry, Depress Anxiety. Additional relevant RCTs will be obtained by hand-searching reference lists of included studies and relevant reviews. We will also contact corresponding authors of included RCTs, manufactures, FDA, and other possible institutions for unpublished trials.	8-9
Search strategy	10	[depress* or dysthymi* or mood disorder* or affective disorder*], combined with [adolesc* or child* or boy* or girl* or juvenil* or minors or paediatri* or pediatri* or pubescen* or school* or student* or teen* or young or youth*], and combined with a list of antidepressants, including [selective serotonin reuptake inhibitors or SSRI or fluoxetine or fluvoxamine or paroxetine or sertraline or citalopram or escitalopram or serotonin and norepinephrine reuptake inhibitors or SNRI or duloxetine or venlafaxine or desvenlafaxine or milnacipran or levomilnacipran or mirtazapine or mianserin or nefazodone or trazodone or vortioxetine or vilazodone or bupropion or reboxetine or agomelatine].	9
Study records:			
Data management	11a	We will first manually remove duplicates of initial search results. We will screening citations based on the inclusion and exclusion criteria.	9
Selection process	11b	Then two experienced reviewers will independently screen titles and abstracts from the retrieved results for possible candidates. We will exclude the trials in which both reviewers judge they do not meet eligibility criteria. Full texts of all remaining papers will be retrieved, and two reviewers will independently examine whether to include them by the same eligibility criteria. Any difference of opinion, for each step, between the reviewers will be resolved through discussion with another member of the reviewing team, or by contacting the authors of the trials for clarification. The selection process of retrieved studies and the reasons for exclusion of trials (e.g. ineligible populations, not randomized trials) will be shown in a flow chart.	9
Data collection	11c	From the included RCTs, two reviewers will independently extract the trial level information using standardized data collection	9-10

For peer review only

process		forms, including trial characteristics, patient characteristics, intervention details, and any other information relevant to this review. We will contact all corresponding authors of included RCTs and ask for their cooperation in this project. The corresponding authors' contact information will be abstracted from the papers, online research profiles (e.g., Google Scholar), or other available ways. Specifically, we will (1) send emails to the authors explaining the study purpose, and invite them to cooperate in this project; (2) send reminder emails 4 and 6 weeks later if no response; (3) contact the corresponding authors by phone or possible personal contacts.	
Data items	12	Trial level information and individual participant data to be obtained from the original authors are shown in Table 1, respectively.	10, 18
Outcomes and prioritization	13	Types of outcome measures<br>Primary outcomes<br>(1) Overall efficacy<br>The primary outcome of efficacy will be the overall change in depressive symptoms, as measured using Children's Depression Rating Scale Revised (CDRS-R) from baseline to endpoint. For RCTs which didn't measure CDRS-R, we will try to convert other depression scales (such as HAMD29 or MADRS30) scores to CDRS-R scores, by using a factor derived from the RCTs that used both scales.<br>As shown in our previous network meta-analysis,13 trial duration varied from 6 weeks to 36 weeks, and the majority of trials employed a treatment duration of 8 weeks. We will try to obtain repeated measures from individual trials if possible. To improve comparability between the included trials, we will prefer the data from 8-week (or the closest to 8-week) time point for efficacy outcomes.<br>(2) Overall tolerability<br>The tolerability of treatment will be the proportion of patients who drop out of the trials early due to side effects at the end of the blinded treatment.<br><br>Secondary outcomes<br>(1) Response rate<br>Response rate will be defined as 50% reduction from baseline to endpoint on CDRS-R (or another standardised rating scale such as HAMD or MADRS).<br>(2) Remission rate<br>Remission rate will be defined as the CDRS scores of less than 28.31<br>(3) Deterioration rate<br>Deterioration represents the depression symptom severity increases after treatment. Deterioration rate will be defined as the proportion of patients whose CDRS-R scores from baseline to endpoint had reliable change index below the cut-off of −1.96.32<br>(4) Overall acceptability<br>The acceptability of treatment will be the proportion of patients who drop out of the trials early for any cause at the end of the blinded treatment.<br>(5) Suicide-related outcomes<br>Suicide-related dichotomous and continuous outcomes will be measured. We will extract the number of participants with suicide-related events (combined suicidal ideation and suicidal behavior) during the acute treatment, as measured on a standardised,	7-8

For peer review only

		validated and reliable rating scale, or reported cases of suicidal ideation and behavior.33 In addition, if data are available, we will also collect data on suicidal ideation as a continuous outcome where a standardised, validated, and reliable rating scale, such as the Suicidal Ideation Questionnaire-Junior High School version (SIQ-JR),34 has been used.<br>(6) Global functioning<br>The outcome of global functioning will be the overall change in validated scales from baseline to endpoint. The commonly used tools of functioning scales included the Children's Global Assessment Scale (CGAS),35 Global Assessment of Functioning (GAF),36 etc.	
Risk of bias in individual studies	14	Two independent review authors will use the Cochrane Collaboration's Risk of bias' tool39 to evaluate the methodological and hence bias risk of eligible studies, and quality assessment will be reported on a study level. The risk of bias will be assessed across seven items, including random sequence generation, allocation concealment, blinding of intervention, blinding of outcome assessment, incomplete outcome data, selective outcome reporting, and other bias, with three level of risk (high, unclear, low). We will rate the quality of study as follows: high risk study (2 or more items rated as high risk of bias); low risk study (5 or more items rated as low risk and no more than one as high risk); unclear risk study (all remaining situations). Any disagreements will be resolved by consensus or consulting the original authors.	10
Data synthesis	15a	All analyses will be performed by intention-to-treat (ITT) analysis. Descriptive statistics will be presented as mean (SD) or median (IQR) for continuous variables and number (percent) for categorical variables.	11
	15b	Individual patient data meta-analyses<br>We will first use the one-stage approach to perform the IPD-MAs, as it offers the highest degree of flexibility for making necessary assumptions, and uses a more exact statistical approach than two-stage approach. We will perform analyses in Stata with the commands mixed (for linear random-effects models), meqrlogit (for logistic models) and ipdforest (for forest plot). To account for between study differences, we will use mixed-effects logistic models for categorical outcomes, and mixed-effects linear regression models for continuous outcomes. Treatment assignment will be introduced as a fixed-effects variable "treatment". As outcomes might vary across studies, we will force the "study" and the interaction term "study*treatment" as random-effects variables into all models. The important clinical and demographic predictors variables (e.g., sex, age, baseline severity score, and treatment duration) will be used as regressors in the models. The heterogeneity of treatment effects across studies will be assessed using the I^2 statistic.	11
Meta-bias(es)	16	We will use contour enhanced funnel plot to detect publication bias for study level data (full set of studies meeting inclusion criteria) and patient level data (the set of studies that were included in the IPD-MA), if at least ten studies are available. We will also use Egger's test to quantify the bias, with a *P* value < 0.10 taken to indicate statistical evidence of asymmetry. In order to examine the effects of non-participation of eligible studies, we will conduct a meta-regression analysis with the effect size of primary outcomes (based on study level data) as the dependent variables, and whether or not the patient level data are included as the predictor indicating. The analyses will be conducted in Stata version 14.0.	10-11
Confidence in cumulative evidence	17	NA.	NA

*** It is strongly recommended that this checklist be read in conjunction with the PRISMA-P Explanation and Elaboration (cite when available) for important clarification on the items. Amendments to a review protocol should be tracked and dated. The copyright for PRISMA-P (including checklist) is held by the PRISMA-P Group and is distributed under a Creative Commons Attribution Licence 4.0.**

For peer review only

From: Shamseer L, Moher D, Clarke M, Ghersi D, Liberati A, Petticrew M, Shekelle P, Stewart L, PRISMA-P Group. Preferred reporting items for systematic review and meta-analysis protocols (PRISMA-P) 2015: elaboration and explanation. BMJ. 2015 Jan 2;349(jan02 1):g7647.

For peer review only
